# The Role of the Nrf2/ARE Antioxidant System in Preventing Cardiovascular Diseases

**DOI:** 10.3390/diseases4040034

**Published:** 2016-11-11

**Authors:** Robert E. Smith, Kevin Tran, Cynthia C. Smith, Miranda McDonald, Pushkar Shejwalkar, Kenji Hara

**Affiliations:** 1 US Food & Drug Administration, 11510 W 80th Street, Lenexa, KS 66214, USA; kevin.tran@fda.hhs.gov (K.T.); Cynthia.smith@fda.hhs.gov (C.C.S.); Miranda.Mcdonald@fda.hhs.gov (M.M.); 2 Department of Applied Chemistry, School of Engineering, Tokyo University of Technology, 1404-1 Katakuramachi, Hachioji, Tokyo 192-0982, Japan; pshejwalkar2004@gmail.com (P.S.); haraknj@stf.teu.ac.jp (K.H.)

**Keywords:** Nrf2, transcription, antioxidants, EGCG, resveratrol, cardiovascular diseases, multi-drug resistant cancer

## Abstract

It is widely believed that consuming foods and beverages that have high concentrations of antioxidants can prevent cardiovascular diseases and many types of cancer. As a result, many articles have been published that give the total antioxidant capacities of foods in vitro. However, many antioxidants behave quite differently in vivo. Some of them, such as resveratrol (in red wine) and epigallocatechin gallate or EGCG (in green tea) can activate the nuclear erythroid-2 like factor-2 (Nrf2) transcription factor. It is a master regulator of endogenous cellular defense mechanisms. Nrf2 controls the expression of many antioxidant and detoxification genes, by binding to antioxidant response elements (AREs) that are commonly found in the promoter region of antioxidant (and other) genes, and that control expression of those genes. The mechanisms by which Nrf2 relieves oxidative stress and limits cardiac injury as well as the progression to heart failure are described. Also, the ability of statins to induce Nrf2 in the heart, brain, lung, and liver is mentioned. However, there is a negative side of Nrf2. When over-activated, it can cause (not prevent) cardiovascular diseases and multi-drug resistance cancer.

## 1. Introduction

**Figure 1:**
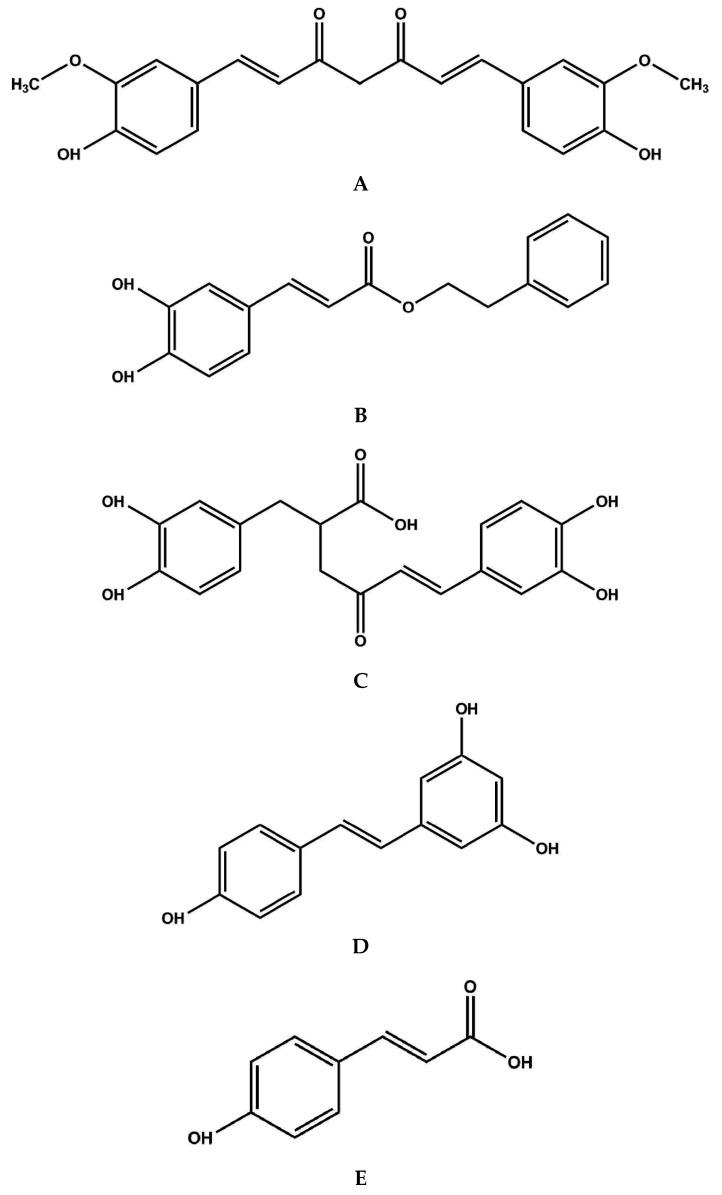
Structures of curcumin (**A**); caffeic acid phenethyl ester (CAPE) (**B**); rosamarinic acid (**C**); resveratrol (**D**) and *p*-coumaric acid (**E**).

Foods and beverages that have high concentrations of antioxidants (such as phenolic compounds) can help prevent cardiovascular diseases and many types of cancer [1]. In the past, it was thought that dietary antioxidants exerted their health benefits by reacting with reactive oxygen species (ROS) as well as reactive nitrogen species and destroying them [2]. This led to the development and use of several in vitro assays to measure antioxidant capacities [3]. Moreover, many foods and beverages (such as green tea) have been called “super-foods”, due to their high in vitro antioxidant capacities [4]. However, there is now much evidence to refute the hypothesis that dietary antioxidants act in vivo by reacting directly with ROS [5]. Instead, several specific compounds exert their health effects by activating antioxidant (and other) genes that are controlled by promoter regions that control their expression. Once activated, nuclear erythroid-2 like factor-2 (Nrf2) binds to the endogenous antioxidant response elements (AREs) that are DNA sequences that respond to dietary antioxidants. They are in the regulatory regions of various genes [6,7,8,9,10]. This should not be confused with androgen response elements, which are quite different. They respond to the hormone, androgen and do not involve Nrf2. In many cases, AREs are activated by the nuclear erythroid-2 like factor-2 (Nrf2), which is a transcription factor [7,8,9]. So, the Nrf2 signaling system is often called the Nrf2-ARE or Nrf2/ARE signaling system. It controls the expression of many antioxidant and detoxification genes, by binding to antioxidant response elements (AREs) that are DNA sequences that respond to dietary antioxidants. They are in the regulatory regions of various genes [10]. Some of the dietary compounds that have been shown to activate the Nrf2/ARE signaling system are listed in Table 1. The structures of five of them that are phenolic compounds with similar structures are shown in Figure 1.

Some of these compounds deserve more elaboration. For example, cyanidin and cyanidin-3-*O*-glucoside are anthocyanins that have been reported to activate the Nrf2 system [10,27]. However, it was not the intact anthocyanins, but a metabolite produced by gut bacteria, phloroglucinol aldehyde, that activated the Nrf2/ARE system in one study [27]. Protocatechuic acid, cyanidin-3-*O*-glucoside, syringic acid, vanillic acid and gallic acid did not activate the Nrf2/ARE system by themselves. So, one’s ability to activate the Nrf2/ARE system by consuming dietary anthocyanins and other phenolic compounds may depend on having a healthy gut microbiome [27].

There are also many compounds from Asian, African and American fruits, vegetables and natural remedies that activate the Nrf/ARE system [14,30]. This includes extracts of *Withania somnifera* (Ashwagandha), *Sutherlandia frutescens* (Sutherlandia) and *Euterpe oleracea* (açaí) [14]. There is also at least one dietary supplement that appears to activate the Nrf2/ARE signaling system. It is called Protandim^®^ and contains ashwagandha, bacopa extract, green tea extract, silymarin, and curcumin [32]. They appear to act synergistically [33]. This supplement also increased the median lifespan of male mice [34]. However, one should be careful in controlling the doses of compounds and/or supplements that activate the Nrf2/ARE system. It has potentially deadly properties when over-activated [8,9,35,36,37,38,39,40,41,42,43,44,45,46,47]. The Nrf2/ARE system is over-activated in some forms of multidrug-resistant cancer and cardiovascular diseases [8,9,35,36,37,38,39,40,41,42,43,44,45,46,47].

**Figure 2:**
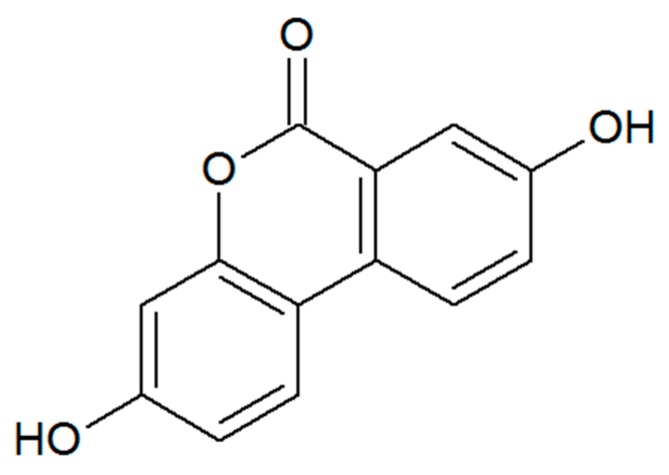
Structure of urolithin A.

It should also be noted that ellagitannins in pomegranates, walnuts, strawberries and other fruits can indirectly activate the AREs and exert many health effects [48,49]. They are converted to urolithin A by select bacteria in the gut. The structure of urolithin A is shown in Figure 2.

However, the ability to produce urolithin A can depend on the status of the gut microbiome [50]. It was produced by gut bacteria in normal rats, but not in rats that had ulcerative colitis [50]. Moreover, urolithin A increased the concentrations of probiotic bifidobacteria and lactobacilli, as well as probiotic strains of *Clostridium* [50]. This prevented the colonization and invasion of colonic tissue by pathogenic enterobacteria [50]. However, the relative concentrations of different species of bacteria in the gut and intestines are different in people who have excess abdominal fat and have metabolic syndrome [51]. The gut microbiome also tends to be healthier in vegetarians and vegans [52,53]. So, ellagic acid and ellagitannins may or may not be metabolized to urolithin A very efficiently, depending on the status of the gut microbiome. So, it is noteworthy that urolithin A is commercially available and that it has multiple health effects [32]. There is also a prescription drug for treating multiple sclerosis (dimethyl fumarate) that acts by inhibiting Nrf2 [54]. It activates the Nrf2 signaling pathway [55], but in the process, it depletes glutathione, decreased cell viability and inhibition of mitochondrial oxygen consumption and the rate of glycolysis in a dose-dependent manner [56]. In contrast, monomethyl fumarate activates the Nrf2 pathway without depleting glutathione. So, it might be the better choice for developing a new drug to treat Parkinson’s disease [56]. In addition, the pharmacodynamics of dimethyl fumarate are tissue specific and involve more genes than just the one that codes for Nrf2 [57]. This includes *Sqstm1* in the kidneys as well as *Osgin1* and *Bdnf* in the brain [57]. Dimethyl fumarate also induces changes in the innate and adaptive immune systems independent of Nrf2 [58]. It also inhibits the nuclear factor NF-κB pathway in breast cancer [59].

There are also some compounds that can inhibit the Nrf2/ARE signaling system, instead of activating it [60,61,62,63,64,65,66,67,68]. Some of them are listed in Table 2. It should be noted that three of them, EGCG, ascorbic acid and luteolin, are listed as both activators (Table 1) and inhibitors (Table 2). This is because they were tested at different concentrations. For example, it took >200 μM EGCG to inhibit the Nrf2/ARE system in human lung adenocarcinoma A549 cells in vitro [63]. It is almost impossible for concentrations of EGCG to ever be so high in vivo. That is, the maximum concentration of EGCG that was found in the blood plasma of human subjects in a pharmacokinetic study was 77.9 ± 22.2 ng/mL, or about 0.17 μM [69]. Even when EGCG is inserted into nanoparticles, its maximum concentration in blood plasma was 704 ng/mL, or 1.5 μM [70]. Similarly, 0.83 μM luteolin was shown to activate the Nrf2/ARE system in hepatocytes that had been exposed to the carcinogenic dioxin, TCDD (2,3,7,8-tetrachlorodibenzodioxin), at a concentration of 0.2 nM [71]. On the other hand, 1 μM luteolin inhibited the Nrf2/ARE system in A549 lung cells in vitro [61]. So, the effect of luteolin may depend on the type of cell to which it is administered. Similarly, the effect of ascorbic acid seems to depend on the type of cells to which it is administered. It activated the Nrf2/ARE pathway in rat RAW 264.7 macrophages when present at concentrations of 10 to 300 μM and increased the survival of endotoxemic mice at a dose of 300 mg/kg, *i.p.* [31]. On the other hand, 125 μM (0.125 mM) ascorbic acid restored the sensitivity of leukemia cells to the anti-cancer drug imatinib by inhibiting the Nrf2/ARE system [58]. In another study, 1000 μM ascorbic acid antagonized the activation of Nrf2/ARE caused by administering resveratrol to hepatocytes in vitro [72]. It specifically antagonized the endogenous antioxidant enzyme, heme oxygenase-1 (HO-1), which is an ARE that is activated by resveratrol [67]. However, resveratrol still exerted its antioxidant effects by activating the antioxidant enzyme called paroxonase, which is not activated by Nrf2 [72]. It should be noted that relatively high concentrations of ascorbic acid in blood plasma can be obtained by consuming high doses of vitamin C as a dietary supplement. That is, when the daily oral dose was increased from 250 to 2500 mg, the concentration in plasma increased from 68 to 85 μM [73]. Moreover, liposomal and intravenous doses of ascorbic acid can produce plasma concentrations up to 400 and 15,000 μM, repsectively [74].

## 2. The Keap1-Nrf2-ARE Signaling System

### 2.1. Overview of Nrf2 Signaling

It is important to maintain a healthy balance in the amount of ROS and the redox state of cells [41,75]. So, the activity of the Nrf2-ARE antioxidant system must be turned on only when it is needed. Its activity is limited by the binding of an inhibitor protein called Keap1 (Kelch-like enoyl-CoA hydratase-associated protein 1) [76]. So, it is sometimes called the Keap1-Nrf2-ARE signaling system. Under conditions of low oxidative stress, Nrf2 is bound to Keap1, which is anchored to actin in the cytoskeleton in the cytosol [7,77]. This complex makes the Nrf2 protein accessible to reaction with the ubiquitous protein called ubiquitin [78]. Ubiquination causes many transcription factors (including Nrf2) to be broken down (hydrolyzed) in subcellular organelles called proteasomes when DNA transcription should not be activated [78]. However, this breakdown of Nrf2 can be prevented by breaking the bonds between it and Keap1 [76,77,78]. That is, the Keap1 protein contains several cysteine residues with sulfhydryl groups that can react with ROS and electrophiles, thus breaking the bonds between Keap1 and Nrf2. Once the bonds are broken, Nrf2 translocates to the cell nucleus, where it can bind to regulatory regions of DNA that turn on the transcription of genes coding for antioxidant elements. These natural antioxidant elements include the enzymes superoxide dismutase (SOD), thioredoxin (TXN), thioredoxin reductase (TXNRD), sulfiredoxin (SRXN), NAD(P)H:quinone oxidoreductase-1 (NQO1), HO-1, glutathione reductase (GR), glutaredoxin (Grx), glutamate cysteine ligase (GCL), glutathione S-transferase (GST), UDP-glucuronyl transferase, thioredoxin reductase, peroxiredoxin sulfotransferase and γ-glutamate cysteine ligase catalytic subunit (GCLC) [30,79,80]. In addition, the expression of over 500 genes is modulated by the Nrf2/ARE pathway [30]. This includes phase I and II detoxfication enzymes, transport proteins, proteasome subunits, chaperones, growth factors and their receptors, as well as some other transcription factors [30].

This is an example of a biological regulatory process that enables metabolism to adapt to changes and the needs of the entire organism [81]. Such regulatory processes require a signal and a sensor to switch-on the adaptive process, a transducer, a modulator of sensitivity, an effector, and a way to switch the signal off. It is also important that such processes communicate (or crosstalk) with other signaling systems [81]. It should be noted that biochemists use the term crosstalk very differently than engineers. In electronics, crosstalk is when a signal transmitted by one circuit (such as a radio frequency transmitter) causes an undesirable effect (such as noise) in the other circuit (such as a radio frequency acceptor). In biochemistry, crosstalk (communication) between signaling pathways is not just advantageous, but absolutely necessary to support life.

Keap1 is the redox sensor of the Keap1-Nrf2 system [81]. The reactive sulfhydryls in the cysteine residues of Keap1 can sense oxidative stress. Once it is released from the cytosolic complex with Keap1, Nrf2 becomes phosphorylated at Ser40, so it can enter the nucleus. Its activity can be decreased or enhanced by activating or inhibiting its export out of the nucleus. If Nrf2 becomes phosphorylated again—this time at Tyr568, it can be exported out of the nucleus. There are nuclear export signals in the leucine zipper domain and transactivation domain of Nrf2. They can be blocked by binding to the musculo-aponeurotic fibrosarcoma (Maf) protein [81].

So, oxidative stress and other primary signals are sensed by Keap1 [81]. They activate AREs, modulated by phosphorylation of Ser40 and Tyr568 and turned off by nuclear export and subsequent destruction of Nrf2 in the proteasome. This is done by ubiquination. Several proteins are required, including a Cullin-3 based ligase (Cul3) that targets the Nrf2 protein in the Keap1-Nrf2 complex. The effectors of the primary signals are the AREs. The AREs are DNA sequences that respond to dietary antioxidants. They are in the regulatory regions of various genes [6,7,8,9,10]. The signals can be turned off by not just nuclear export, but also by other mechanisms. There are also Keap1 proteins in the nucleus. They can bind to Nrf2 and target it for degradation in nuclear proteasomes. The actin cytoskeleton must be polymerized for it to bind to the Keap1-Nrf2 complex. Cellular oxidants can activate the enzyme phosphatidylinositol 3-kinase (PI3K), which depolymerizes the actin. Re-polymerization allows Nrf2 to be exported from the nucleus. Moreover, actin can be covalently modified by the attachment of glutathione. This leads to the de-polymerization of actin. This can be prevented by Grx, which is a small redox enzyme that uses glutathione as a cofactor. The Keap1-Nrf2 signaling system also activates the transcription of DNA coding for proteins like Cul3, Rbx and Keap1 that are cytosolic inhibitors of this system. Finally, there are many enzymes that can eliminate the system’s signals or prevent them from being formed in the first place [81].

### 2.2. Effects on Mitochondria

The Keap1-Nrf2 signaling system is also affected by crosstalk with other signaling systems [81,82]. As mentioned previously, Nrf2 can be phosphorylated and dephosphorylated. This links it with protein kinases and phosphatases. In addition, it is affected by crosstalk with the mitogen-activated protein kinase (MAPK), casein kinase 2, the protein kinase R (PKR)-like endoplasmic reticulum kinase (PERK), protein kinase C, PI3K and its partner, Akt [81]. That is, PI3K catalyzes the biosynthesis of phosphatidylinositol (3,4,5)-trisphosphate or PtdIns(3,4,5)-P_3_, which activates Akt, also known as protein kinase B [82]. It was named Akt because it was first found in a retrovirus called Akt8 [82]. In addition, the tumor suppressor protein p53 has antioxidant functions that include activating the transcription of the gene coding for Nrf2 and the proper maintenance of mitochondria function, which limits the production of ROS [83]. However, p53 and Nrf2 have many different effects on different types of cells and under different physiological conditions. For example, p53 activates the form of regulated cell death called ferroptosis, which Nrf2 inhibits [84]. That is, one of the hallmarks of living organisms and cells is autopoeisis, or self-making [85,86]. Organisms, tissues and cells have an outer layer (skin, epidermis and cell membranes) that is continuously being broken down and re-made, along with many of its inner components. Under stressful conditions, such as a lack of sufficient nutrients, cells can scavenge some of their internal parts in a process called autophagy. When mitochondria are being scavenged, it is called mitophagy. Nrf2 promotes mitophagy and helps maintain mitochondrial homeostasis [87]. On the other hand, ferroptosis, apoptosis (programmed cell death) can occur when it is time for an entire cell to die [84]. Ferroptosis is caused by an excess of poorly liganded iron and ROS, as well as activation of MAPKs, p53 and other signaling systems [84]. That is, some of the iron in our body is bound to hemoglobin, which is a tight ligand [84]. Free, unliganded iron (as Fe^2+^) is frequently consumed as a dietary supplement (FeSO_4_) by premenopausal women, especially when they are pregnant or nursing. It is also consumed by postmenopausal women who are anemic. However, FeSO_4_ is not included in multivitamins for men over 50. This is because poorly liganded iron (Fe^2+^) can react with H_2_O_2_ in the Fenton reaction to produce the hydroxide ion (OH^−^) and highly destructive hydroxyl radical (^•^OH), shown in Equation (1):

Fe^2+^ + H_2_O_2_→Fe^3+^ + OH^−^ + ^•^OH (1)

The hydroxyl radical can oxidize lipids and lead to not just ferroptosis [84], but also atherosclerosis and cardiovascular diseases [85,87]. This has been called the iron hypothesis [85,88]. It helps explain why men are more prone to heart disease than women. It is due to differences in the amounts of stored iron. The amount of stored iron in men increases after adolescence, but remains low in women and only begin to rise after the age of about 45. Non-steroidal anti-inflammatory drugs (NSAIDs) like aspirin that cause gastrointestinal blood loss may protect against heart disease by decreasing iron stores. Moreover, there is a continuous autocatalytic production of hydroxyl radicals involving poorly liganded iron, leading to apoptotic cell death. That is, once Fe^3+^ is produced in the Fenton reaction, Equation (1), it can react with superoxide anions, O_2_^•^^−^, (also produced by mitochondria) to regenerate Fe^2+^ as shown in Equation (2) [85,88].

Fe^3+^ + O_2_^•^^−^ → Fe^2+^ + O_2_ (2)

Once the Fe^2+^ ion is regenerated, it can undergo the Fenton reaction again to produce more of the toxic hydroxyl radical [85,88]. However, if the Fe^2+^ ion is tightly bound (or liganded) to dietary phenolic compounds in fruits and vegetables, it will not undergo the Fenton reaction. So, even the many dietary phenolic compounds that are not listed in Table 1 can help prevent cardiovascular diseases by binding to the Fe^2+^ ion. Still, men over 50 should not take FeSO_4_ as a dietary supplement and should probably limit or stop their consumption of red meat [85,88].

Under conditions of oxidative stress, Nrf2 is activated, along with AREs. They are DNA sequences that respond to dietary antioxidants. They are in the regulatory regions of various genes that decrease the concentrations of ROS [6,7,8,9,10,85]. Nrf2 also increases the synthesis of more mitochondria and protects them from damage by inhibiting the opening of the mitochondrial permeability transition pore and mitochondrial swelling. It also supports the structural and functional integrity of the mitochondria, especially during stressful condition [87].

### 2.3. AMPK Signaling

Nrf2 is affected not only by the p53 protein, but also by several other signal transduction systems [89]. This includes the adenosine monophosphate (AMP) activated kinase (AMPK), which is a central hub in the network that controls cellular energy homeostasis. It decreases anabolism and increases catabolism, improves endothelial function, reduces inflammation, and improves redox balance. Moreover, AMPK works and communicates with (crosstalks) with the Nrf2 system to protect cells from damage caused by unfolded proteins. That is, when a protein is unfolded from its active, folded structure, it is supposed to be marked for degradation (proteolysis). When unfolded proteins are not completely destroyed, they can aggregate and accumulate in the endoplasmic reticulum (ER). This causes an unfolded protein response (UPR) that counteracts ER stress. AMPK and Nrf2 interact to support the UPR and prevent cardiovascular diseases. Tight cooperation between AMPK and Nrf2 controls cellular redox, energy and protein homeostasis [89].

Normal vascular function is important for cardiovascular health. This requires continual turnover of proteins, which is done in proteasomes [78]. Protein turnover is needed to help regulate signaling cascades by controlling the concentrations of transcription factors. It also allows damaged proteins to be replaced, thus preventing cellular oxidative damage. Proteasomal dysfunction in aging and atherosclerosis may cause vascular dysfunction. This can prevent proteasomes from removing oxidized proteins, producing large protein aggregates. They are extensively cross-linked and can be further modified by advanced glycation end products, lipid peroxides or ubiquitin, preventing protein unfolding and consequently degradation by the proteasome. Moreover, protein aggregates can inhibit proteasomal activity directly [78].

Nrf2 also interacts with a protein deglycase called DJ-1 and Parkinson disease protein 7 [90]. It protects neurons from oxidative stress and aggregation of the protein α-synuclein, which can lead to Parkinson’s disease. DJ-1 also acts as a natural antioxidant by activating the Nrf2/ARE system. It does this by binding to Keap1, preventing it from inhibiting Nrf2 [90].

### 2.4. Notch Signaling

Nrf2 also interacts and crosstalks with the Notch signaling pathway [91]. The Notch signaling pathway influences the cell cycle as well as cellular differentiation, survival, proliferation and apoptosis (programmed cell death). It transduces primary signals at the cell membrane of target cells. It goes into the nucleus to activate the expression of several genes. The exact responses depend on the type of cells and their needs. The Notch pathway exerts pleiotropic effects in each tissue that expresses the Notch protein. Thus, Notch-signaling networks regulate various events in embryonic and postnatal development. Like the Nrf2 signaling system, Notch is conserved from worms (*Caenorhabditis elegans*) to humans. They can be regulated by reciprocal transcription. That is, Notch1 targets the expression of the gene coding for Nrf2 and Nrf2 targets Notch expression. Nrf2–Notch crosstalk protects against endogenous and exogenous stressors by activating the expression of defense systems. This leads to cytoprotection, while maintaining cellular homeostasis and tissue organization. These effects may vary between different tissues and within specific regions, such as the niche where adult tissue stem cells or progenitor cells reside [91].

Even though the Keap1-Nrf2-ARE signaling system exists in so many animals, the level of its activity is quite variable [92]. It is much more active in the relatively long-lived naked mole-rat (*Heterocephalus glaber*) than in other rodents with shorter lifespans. Moreover, species that live longer are more resistant to both chronic and unpredictable stressors. They are also more resistant to age-related diseases, including cardiovascular diseases. However, it is not the concentration of Nrf2 itself that controls its total cellular activity. Instead, it is the concentrations of Keap1 and the β-transducin repeat containing protein (βTrCP), both of which target cytosolic Nrf2 for proteolytic destruction. So, it was suggested that βTrCP could be a good therapeutic target. It is conserved in mice, mole-rats and humans. It could be a better target than Keap1, since low concentrations of it produce fewer harmful side effects than those caused by low levels of Keap1 [92].

However, the βTrCP protein does not act in isolation [93]. As mentioned previously, phosphorylation of serine residues in Nrf2 enable it to dissociate from the complex with Keap1 and enter the nucleus. There are several protein kinases that can catalyze this phosphorylation. They include protein kinase C, protein kinase RNA-like endoplasmic reticulum kinase (PERK), casein kinase 2, the SRC (sarcoma) family of protein kinases and glycogen synthase kinase-3 (GSK-3). In addition, the PI3K-Akt signaling system induces the expression of one of the genes coding for an ARE, HO-1. The PI3K-Akt signaling system also enables Nrf2 to sustain cell proliferation by reprograming glucose and glutamine metabolism. It does this by first targeting glycogen synthase kinase-3 (GSK-3). GSK-3 catalyzes the phosphorylation of the SRC-related kinase, FYN. This tyrosine kinase is translocated to the nucleus, where it catalyzes the phosphorylation of Nrf2 at Tyr568. This targets the phosphorylated Nrf2 for nuclear export and degradation in the cytosol. The β-TrCP protein recognizes phosphorylated Nrf2 and targets it for ubiquination and proteolysis. So, Keap1 and β-TrCP have been described as limiter and regulator valves, respectively. They control the movement of Nrf2 in and out of the nucleus of the cell. Under normal redox homeostasis and the absence of stimulation by a growth factor, they both act to limit the flow of Nrf2 into the nucleus. Under normal redox homeostasis but in the presence of signaling by a growth factor, the Keap1 “valve” stays closed while the β-TrCP opens to release a small percentage of the Nrf2 for entry into the nucleus. During both redox imbalance and receptor signaling, both the Keap1 and β-TrCP “valves” open the flow of Nrf2 into the nucleus. This combination is unlikely under normal physiological conditions, but could be caused by pharmaceutical intervention. That is, drugs might be developed that could reduce the concentration of Nrf2 by targeting the GSK-3/β-TrCP system [93].

## 3. The Role of the Keap1-Nrf2-ARE Signaling System in Cardiovascular Diseases

### 3.1. Overview

The Keap1-Nrf2-ARE signaling system can prevent cardiovascular disease (CVD) by preventing smoldering inflammation. It does this by activating the natural antioxidant systems in cells. Smoldering inflammation is a chronic, relatively low level of inflammation that is caused by ROS and reactive nitrogen compounds [82]. The heart requires much energy that is produced mostly by mitochondrial oxidative phosphorylation [6]. It consumes more energy than any other organ. Even when resting, it uses about 8–15 mL O_2_/min/100 g heart. This can increase to as much as 70 mL/min/100 g heart when exercising vigorously. Every day the adult heart beats about 100,000 times, pumping approximately 10 t of blood throughout the body, and recycling around 6 kg of ATP (20–30 times its own weight). The ROS and reactive nitrogen compounds produced as by-products can cause inflammation if they are not destroyed effectively [6].

### 3.2. Inflammation Is Misunderstood in the Past

However, inflammation is a topic that has been misunderstood in past scientific articles and continues to be misunderstood by many consumers. That is, many scientific articles have been published that give the total antioxidant capacities of foods in vitro. Also, some foods, like açaí have been called superfoods because they have relatively high antioxidant capacities. The U.S. Department of Agriculture even had a website for a few years that listed the in vitro antioxidant capacities of many foods and spices [82]. However, they removed the data from their website because there is no evidence that consuming large quantities of antioxidants have any preventive or therapeutic effects [82]. Moreover, the idea that dietary antioxidants act by reacting directly with ROS in vivo is largely discredited [5,94]. Instead, some of them alter cell signaling and mitochondrial function through the Keap1-Nrf2-ARE signaling system [30,60,61,62,63,64,65,66,67,68].

### 3.3. Inflammation Is Important in CVD

Still, inflammation plays an important role in atherosclerosis and the emergence of CVD [82,85,86,87,88]. Atherosclerosis is the main process underlying CVD. It starts when endothelial cells that line the intima are activated by saturated fatty acids and/or cholesterol. It leads to the expression of adhesion proteins on leukocytes, making them bind to the endothelium. Once they are bound, they can migrate through the endothelium to the intima where they can attract monocytes that can change into lipid-laden foam cells. This process is often enhanced in people who have type-2 diabetes. After immune cells and inflammatory mediators interact, atheroma can emerge and rupture-prone atherosclerotic plaques are made. Pro-inflammatory signaling pathways are also involved in thrombosis, the late stage of atherosclerosis. It is responsible for most of the clinical complications of CVD. So, CVD can be a major consequence of obesity-induced inflammation and type-2 diabetes [82,85,86,87,88].

Inflammation is more important than cholesterol concentrations in causing CVD [82]. About half of all heart attacks and strokes occur in people with normal or even low concentrations of cholesterol in their blood. Normal, healthy endothelial cells (ECs) on the innermost surface of arterial walls are able to resist adhesion by leukocytes [95]. When a person smokes tobacco, consumes many saturated fats, is hyperglycemic or resistant to insulin, and has metabolic syndrome with high blood pressure, adhesion molecules are expressed by ECs [82]. This enables leukocytes to attach to the arterial wall. VCAM-1 then binds to monocytes and T lymphocytes, which are found in early atherosclerotic plaques. This can be prevented by laminar blood flow, which activates some anti-atherosclerotic mechanisms. This includes the expression of the natural anti-oxidant, superoxide dismutase and an increase in nitric oxide (NO) synthase [82]. There is also an increase in the concentration of NO, which causes vasodilation and limits the expression of the gene coding for VCAM-1 [82,95].

### 3.4. The Keap1-Nrf2-ARE System Can Sense Shear Stress

So, it is important to note that the Keap1-Nrf2-ARE system can sense shear stress in ECs and protect against vascular dysfunction and atherosclerosis [96]. Nrf2 is highly sensitive to laminar fluid shear stress, which interacts with the epithelium to maintain vascular homeostasis. This is done by linking biomechanical forces with signal transduction to maintain a balance in the redox state. ECs respond to changes in shear stress to modulate redox signaling. This leads to changes in the expression of AREs, the inflammatory phenotype and cell alignment as well as structural remodeling of blood vessels. Also, when ECs are exposed to oscillatory disturbed shear (OS) forces, the expression of histone deacetylases (HDACs) is induced. One of the HDACs catalyzes the deacetylation of Nrf2, thus decreasing its activity. There is another important epigenetic mechanism that affects Nrf2 activity. Redox-sensitive microRNAs (miRNAs) can modulate the concentrations of Nrf2 and some of the regulators of Nrf2 signaling. The expression of these miRNAs is different in laminar compared oscillatory fluid shear stress. Thus, there are several mechanisms by which Nrf2 can react to fluid shear stress and help prevent CVD [96].

### 3.5. The Nrf2 System Protects Mitochondria

Another way that Nrf2 helps to prevent CVD is by protecting mitochondria from oxidative stress [87,97,98,99,100]. Cardiomyocytes have more mitochondria than any other type of cell [99]. However, they produce H_2_O_2_ as a byproduct of oxidative phosphorylation. As mentioned before, the Keap1-Nrf2-ARE signaling system activates the production of natural antioxidants. This includes glutathione, thioredoxin, and NADPH [95]. Nrf2 also upregulates the transcription of genes coding for uncoupling protein 3 (UCP3). Nrf2 influences mitochondrial biogenesis by maintaining the concentrations of nuclear respiratory factor 1 and peroxisome proliferator-activated receptor γ coactivator 1α, as well as by promoting purine nucleotide biosynthesis. When some of the mitochondria become irreparably damaged, Nrf2 stimulates mitophagy [87,100].

In healthy cells, mitochondria exist in elaborate networks and provide cells with ATP by oxidative phosphorylation of nutrients through a series of protein complexes. In this process, protons (H^+^) and electrons are separated [101]. Electron transport is coupled to the active transport of H^+^ across the inner mitochondrial membrane. This electrochemical force is accompanied by a proton gradient that helps make ATP. An electron transport chain is coupled to this proton motive force. However, this process is not completely coupled. Some protons leak through the inner mitochondrial membrane in a process that is mostly controlled by five uncoupling proteins, UCP1—UCP5 [101,102,103]. This decreases the membrane potential and helps to limit the production of excess ROS by mitochondrial complexes I and III [102]. UCP4 and UCP5 are primarily located in neurons [103]. UCP1 is an important adaptor of thermogenesis in brown adipose tissue in mammals [101,102,103]. UCP2 is expressed in white adipose tissue, liver, and cardiac and skeletal muscle, while UCP3 is mostly expressed in brown adipose tissue and skeletal muscle, and at lower levels in cardiomyocytes (cardiac muscle cells) [102]. The expression of UCP2 increases as the concentration of ROS increases, subsequently producing a negative feedback that limits the production of ROS [100]. Both UCP2 and UCP3 help control the production of ROS and the oxidative damage that they can produce in the heart if not properly controlled [10,104]. Nrf2 can protect mitochondria and prevent myocyte death by activating the transcription of UCP2 and UCP3 [102,104].

### 3.6. Nrf2 Also Activates HO-1

Nrf2 also activates HO-1, which prevents apoptosis in cardiomyocytes [98]. That is, when HO-1 is over-expressed, it can produce carbon monoxide (CO), which stimulates SOD and mitochondrial H_2_O_2_ production. This activates protein kinase B (more frequently known as Akt), which activates glycogen synthase kinase 3-β, which allows more Nrf2 to be released from Keap1 and be translocated to the cell nucleus. The accumulation of nuclear Nrf2 opposes apoptosis and necrosis caused by the anti-cancer drug, doxorubicin. Even though higher concentrations of CO are toxic, lower concentrations can be healthy. That is, CO can bind to the reduced *a3* heme of cytochrome *c* oxidase and increase H_2_O_2_ production. Despite its toxicity at higher concentrations, H_2_O_2_ is important in signal transduction and production of more mitochondria [98].

### 3.7. Nrf2 Also Supports the ER

The Keap1-Nrf2-ARE signaling system supports the activity of another subcellular organelle—the endoplasmic reticulum, or ER [105]. In CVD and other diseases, unhealthy changes in cells can lead to ER dysfunction and an abnormal accumulation of unfolded proteins [105]. That is, for proteins to function properly, they must fold into a specific structure and not exist as unfolded, random coils that are not very soluble in the cytosol or cell membranes. The folding process is partly controlled by the ER. However, when the protein-folding capacity of the ER is overwhelmed, it can produce ER stress and cardiac hypertrophy [105]. Moreover, a reduction in blood flow caused by atherosclerotic coronary artery disease and hypoxia can induce ER stress. Transmembrane sensors in the ER detect the accumulation of unfolded proteins. They activate transcriptional and translational pathways that deal with unfolded and misfolded proteins. This is called the unfolded protein response (UPR). When the UPR fails to control the concentrations of unfolded and misfolded proteins in the ER, apoptosis is induced. Interventions against ER stress as well as activation of the Keap1-Nrf2-ARE system reduce myocardial infarct size and cardiac hypertrophy in the transition to heart failure. Finally, activation of the Keap1-Nrf2-ARE system may be important in ischemic preconditioning, in which the heart is subjected to one or more episodes of nonlethal myocardial ischemia-reperfusion before coronary artery occlusion can occur [105].

It should also be noted that statins are administered to patients experiencing myocardial infarctions or CVD [97]. They were developed based on their ability to inhibit the rate limiting step in cholesterol synthesis, catalyzed by 3-hydroxy-3-methylglutaryl coenzyme A reductase. Since being introduced to the clinic, many other healthy effects of statins have been discovered. This includes stabilizing plaque, maintaining endothelial function, anti-inflammatory actions and antioxidant capabilities. Recently, it has been shown that statins can also activate the Keap1-Nrf2-ARE signaling system [97].

### 3.8. The Keap1-Nrf2-ARE Signaling System Is Important in Maintaining the Renewal of Cardiomyocytes

The Keap1-Nrf2-ARE signaling system is also important in maintaining the renewal of cardiomyocytes [6,106]. That is, for many decades, the heart was thought to be a post-mitotic organ. It is now known that some cardiac remodeling can occur—especially during aging. The adult heart contains cardiomyocytes, fibroblasts, endothelial cells, vascular smooth muscle cells and extracellular matrix proteins. Proper heart function depends on the cardiomyocytes and the sarcomeres in them that are formed by contractile proteins. Even though the number of cardiomyocytes virtually does not change in adulthood, the amount of sarcomeres does. The change is variable and can be modified as an adaptive response to stress conditions. These can lead to many biochemical and functional changes, such as alterations in Ca^2+^ handling, signaling cascades and energy metabolism. However, cardiomyocytes become more susceptible to oxidative stress during the aging process, resulting in necrotic and apoptotic cell death. The decrease in the amount of cardiomyocytes and their functional causes age-related changes in hearts that are also associated with augmented remodeling processes. This causes an increased heart size, a change from elliptical to spheroid shape, left ventricle wall thickening and increased systolic pressure. Cardiac remodeling in the elderly is often accompanied by interstitial and perivascular fibrosis, thickening of coronary vessels and increased calcification in the myocardium. The loss of functional cells can decreased the regenerative activity of the heart from 1% per year at 20 years of age to 0.4% at 75 years. A decrease in the number of sinoatrial node cells is also associated with aortic stenosis development in 2% of aged adults. This can lead to attenuated diastolic function and cardiac output, decreased maximum stroke volume, lower circulating blood volume and increased arterial stiffness. However, Nrf2 can help to protect against CVD. On the other hand, age-related deregulation of the Keap1-Nrf2-ARE signaling system causes an increase in oxidative stress in cardiomyocytes and the vascular system. So, specific compounds (Table 1) that activate this signaling system may help prevent CVD, type-2 diabetes, renal failure and neurodegenerative diseases [6,106].

One of the best ways to prevent CVD is by exercising regularly. Even though exercising causes a temporary increase in ROS, it is still quite healthy. That is, there is a hormetic physiological response to ROS. Hormesis is when there is a non-linear dose-response curve that is often U-shaped in toxicokinetic studies [85]. At low concentrations, ROS can be healthy, so the toxicity (response) decreases at the lower doses of ROS. However, at higher doses, ROS toxicity increases. For lower doses of ROS to be healthy, the Keap1-Nrf2-ARE system must be functioning properly. In mice that did not have this (Nrf2^−/−^), the genes coding for proteins that regulate the redox state of the cell and ubiquination of misfolded proteins were not regulated properly. This led to autophagy and atrial hypertrophy. In contrast, the hearts of mice that contained active genes coding for Nrf2 (wild-type or Nrf2^+/+^) did recover from stress caused by high intensity exercise [107].

However, like so many things in life, the Keap1-Nrf2-ARE signaling system must be balanced. That is, even though it may be quite healthy to activate it to a limited extent by consuming dietary antioxidants at the concentrations that they occur in foods, it should not be over-activated [6,8,35,42,43,47,56,108,109,110]. For example, some ROS are needed to help control normal insulin signal transduction and glucose-stimulated insulin secretion in pancreatic β-cells. So, persistent activation of the Keap1-Nrf2-ARE system can prevent the required ROS signaling. Some of the detrimental effects of overactive Keap1-Nrf2-ARE signaling include worsening insulin resistance, impairing lipid accumulation in adipose tissue, and increasing hepatic steatosis in leptin-deficient mice. In addition, some oxidative modification of proteins is needed for proper ubiquitination and protein degradation. If the Keap1-Nrf2-ARE system is over-activated, it can decrease necessary protein oxidation, chronic reducing stress, deubiquitination and downstream protein degradation pathways. This can cause cardiac hypertrophy and remodeling [108]. Reduction stress can occur when there is an imbalance between oxidants and antioxidants, in favor of the latter [43]. It was originally defined as an excess of NADH, but now is known to include other reducing agents, such as NADPH and reduced glutathione. An excess of biochemical reducing agents can lead to damaged lipid membranes, deposition of triacylglycerides (triglycerides), mitochondrial dysfunction, cytotoxicity, cardiac ischemic injury and an increased risk of Alzheimer’s disease [43].

### 3.9. Nrf2 in Multi-Drug Resistant Cancer

Finally, multi-drug resistant cancers often have an overactive Keap1-Nrf2-ARE signaling system [55,102,111]. So, even though the lower concentrations of dietary antioxidants that are present in green tea as well as many fruits and vegetables may help prevent CVD and cancer, the much higher concentrations and doses in dietary supplements may help cause multi-drug resistant cancer. Moreover, popular antidiabetic drugs such as the hypoglycemic dipeptidyl peptidase–4 inhibitors (DPP-4i) saxagliptin and sitagliptin, as well as the antineuropathic *a*-lipoic acid (ALA), do not increase tumor incidence but increase the risk of metastasis of existing tumors, based on in vitro and animal models [55]. However, Nrf2 activation from exercise, food, or dietary supplementation in healthy adults appears more likely to be chemopreventive, based on epidemiology and laboratory data [111]. In contrast, prolonged Nrf2 signaling from compromised autophagy is probably a major factor in arsenic-mediated carcinogenesis [111].

## 4. Conclusions

In conclusion, dietary antioxidants do not destroy ROS in vivo by reacting with them directly. Instead, some of them activate the Keap1-Nrf2-ARE signaling system, which controls the expression of many antioxidant and detoxification genes. It binds to antioxidant response elements (AREs) that are DNA sequences that respond to dietary antioxidants. They are in the regulatory regions of various genes [6,7,8,9,10]. This signaling system activates the transcription of several genes that code for natural antioxidants, including the enzymes superoxide dismutase (SOD), thioredoxin (TXN), thioredoxin reductase (TXNRD), sulfiredoxin (SRXN), NAD(P)H:quinone oxidoreductase-1 (NQO1), HO-1, glutathione reductase (GR), glutaredoxin (Grx), glutamate cysteine ligase (GCL), glutathione S-transferase (GST), UDP-glucuronyl transferase, thioredoxin reductase, peroxiredoxin sulfotransferase and γ-glutamate cysteine ligase catalytic subunit (GCLC) [30,74,75]. In addition, the expression of over 500 genes is modulated by the Nrf2/ARE pathway [30]. This includes phase I and II detoxfication enzymes, transport proteins, proteasome subunits, chaperones, growth factors and their receptors, as well as some other transcription factors [30]. Moreover, the Keap1-Nrf2-ARE system protects mitochondria in cardiomyocytes. However, when over-activated, this same system can cause CVD and multi-drug resistant cancers. So, even though the relatively low concentrations of dietary antioxidants in fruits, vegetables and spices might be healthy, higher concentrations might not. Dietary supplements such as green tea extract and purified EGCG might over-activate the Keap1-Nrf2-ARE signaling system and be quite unhealthy.

## Figures and Tables

**Table 1:** Partial list of dietary compounds that have been shown to activate the Nrf2/ARE signaling system.

Compound	Dietary Sources ^a^	References
Epigallocatechin-3-gallate (EGCG)	Green tea	[11]
Curcumin	Turmeric	[12]
Carnosol	Rosemary	[12]
Zerumbone	Ginger	[12]
Caffeic acid phenethyl ester (CAPE)	Honeybee propolis and many plants	[12]
Ethyl ferulate	Many plants, including eggplant	[12]
Sulphorane	Broccoli and other cruciferous vegetables	[12]
Resveratrol	Red wine, Itadori tea	[13]
Quercetin	Many foods, including capers	[14]
Cyanidin and cyanidin-3-*O*-glucoside	Many types of fruits and berries	[10]
Catechin	Many foods, including cocoa and tea	[15]
Epicatechin	Many foods, including cocoa and tea	[16]
Kaempferol	Many foods, including green tea and berries	[16]
Naringenin-7-*O*-glucoside	Many foods, including tomatoes	[16]
Procyanidin B2	Many foods, including cocoa and grape juice	[16]
Genistein	Soybeans	[16]
Butein and phloretin	Fruits, vegetables, nuts, tea, coffee, red wine	[16]
Xanthohumol	Comon hop (*Humulus lupulus*)	[16]
Luteolin	Many foods, including celery and broccoli	[17]
Tangeretin	Tangerines and other citrus fruits	[17]
Ellagic acid	Pomegranates	[18]
Oleanolic acid	Many plants, including olive leaves	[19]
Ganodermanondiol	Lingzhi mushrooms	[20]
Echinatin	Licorice	[21]
Chlorogenic acid	Green coffee extract, coffee	[22]
*N*-methylpyridinium	Coffee	[22]
Ursolic acid	Apple peels and many other foods and spices	[23]
Hydroxytyrosol	Olive oil and olive leaves	[24]
Rosmarinic acid	Rosemary	[25]
Protocatechuic acid	Raspberries and many other foods	[26]
Phloroglucinol aldehyde	Metabolite of anthocyanins	[27]
*p*-coumaric acid	Many foods, including peanut and tomatoes	[28]
Ferulic acid	Many herbs used in traditional Chinese medicine	[29]
Isoorientin	Açaí, passion fruit, *Sasa borealis*	[16,30]
Ascorbic acid	Vitamin C, citrus fruits	[31]

^a^ Only some of the main dietary sources are listed.

**Table 2:** Partial list of dietary compounds that have been shown to inhibit the Nrf2/ARE signaling system.

Compound	Dietary Sources ^a^	References
Ferulic acid	Many plant seeds and cell walls, including *Ferula foetida*	[30,55]
Luteolin	Many foods, including celery and broccoli	[30,56]
EGCG	Green tea and green tea extract	[30,57]
Ascorbic acid	Vitamin C and citrus fruits	[30,58]
Apigenin	Fruits, vegetables	[30,59]
All-*trans*-retinoic acid	From β-carotene	[30,60]
Brusatol	*Brucea javanica*	[30,61]
Trigonelline	Fenugreek seeds	[30,62]
Ochratoxin A	*Aspergillus*, *Penicillum*	[30,63]

^a^ Only some of the main dietary sources are listed.

## Abbreviations

The following abbreviations are used in this manuscript:

AMP	Adenosine monophosphate
AMPK	Adenosine monophosphate kinase
ARE	Antioxidant response element
ATP	Adenosine triphosphate
βTrCP	β-transducin repeat containing protein
CAPE	Caffeic acid phenethyl ester
Cul3	Cullin-3 based ligase
CVD	Cardiovascular disease
DJ-1	Protein deglycase
ECs	endothelial cells
EGCG	Epigallocatechin-3-gallate
ER	Endoplasmic reticulum
GCL	Glutamate cysteine ligase
GR	Glutathione reductase
Grx	Glutaredoxin
GSK-3	Glycogen synthase kinase-3
GST	Glutathione S-transferase
HDACs	histone deacetylases
HO-1	Heme oxygenase-1
Keap1	Kelch-like enoyl-CoA hydratase-associated protein 1
Maf	Musculo-aponeurotic fibrosarcoma protein
MAPK	mitogen-activated protein kinase
miRNA	microRNA
NAD(P)H	Nicotinamide adenine dinucleotide reduced form, with or without a phosphate
NO	nitric oxide
NQO1	NAD(P)H:quinone oxidoreductase-1
Nrf2	Nuclear erythroid-2 like factor-2
Nrf2^+/+^	The wild type of Nrf2, in which both copies of the gene coding for Nrf2 are present
Nrf2^−/−^	The genotype in which neither of the genes coding for Nrf2 are present
OS	oscillatory disturbed shear
p53	A tumor suppressor protein with a molecular weight of 53 kiloDaltons
PERK	Protein kinase RNA-like endoplasmic reticulum kinase
PI3K	Phosphatidylinositol (3,4,5)-trisphosphate
PKR	Protein kinase R
PtdIns(3,4,5)-P_3_	Phosphatidylinositol (3,4,5)-trisphosphate
RNA	Ribonucleic acid
ROS	Reactive oxygen species
SOD	Superoxide dismutase
SRC	Sarcoma
SRXN	Sulfiredoxin
TCDD	2,3,7,8-tetrachlorodibenzodioxin
TXN	Thioredoxin
TXNRD	Thioredoxin reductase
µM	micromoles per liter
UPR	Unfolded protein response
VCAM-1	Vascular adhesion molecule-1
